# Highly sensitive detection of dengue biomarker using streptavidin-conjugated quantum dots

**DOI:** 10.1038/s41598-021-94172-x

**Published:** 2021-07-26

**Authors:** Linh Tran, Sangkwon Park

**Affiliations:** Department of Chemical and Biochemical Engineering, Dongguk University, Pildong-ro 1-gil 30, Jung-gu, Seoul, 04620 South Korea

**Keywords:** Other nanotechnology, Quantum dots, Fluorescent probes

## Abstract

A highly sensitive immunosensor using streptavidin-conjugated quantum dots (QDs/SA) was developed to detect dengue biomarker of non-structural protein 1 (NS1) at very low concentration, so that it can probe dengue infection even in the early stage. The QDs/SA were first bound to biotinylated NS1 antibody (Ab) and the QDs/SA-Ab conjugates were then used to detect the NS1 antigen (Ag) in the Ag concentration range of 1 pM to 120 nM. The formation of QDs/SA-Ab and QDs/SA-Ab-Ag conjugates was confirmed by the measurements of field emission scanning electron microscopy (FF-SEM), field emission transmission electron microscopy (FE-TEM), dynamic light scattering (DLS), and zeta-potential. Fluorescence emission spectra of QDs/SA-Ab-Ag conjugates showed that the magnitude of fluorescence quenching was linearly proportional to the NS1 Ag concentration and it nicely followed the Stern–Volmer (SV) equation in phosphate buffer solution. However, in human plasma serum solution, the fluorescence quenching behavior was negatively deviated from the SV equation presumably due to interference by the serum component biomolecules, and it was well explained by the Lehrer equation. These results suggest that the current approach is promising because it is highly sensitive, fast, simple, and convenient, and thus it has a potential of application for point-of-care.

## Introduction

For the past 60 years, dengue virus infection has been a significant global health issue in tropical and sub-tropical countries around the world^1^. This dangerous viral infection is predicted to expand and continue to threaten more lives as results of global warming, climate change, urbanization, and lack of vector control and public health care system^2^. The annual number of dengue virus infections was estimated to be 390 million using a digital map in 2013^3^ and it has been dramatically increased by a factor of 30 for the last five decades^4^. Dengue fever is a typical mosquito-borne disease. Although the disease causes mild or self-managed symptoms in some cases^5^, many cases have been reported to be severe by causing serious illness and even death. Therefore, this disease demands early diagnosis and proper medical attention and its early detection can contribute to lowering fatality rates significantly less than 1%^6^.

Dengue virus is classified into four serotypes (DENV-1 ~ 4) with a lipopolysaccharide envelop, three structural proteins, a positive single-strand RNA genome, and seven non-structural proteins (NS1, NS2a, NS2b, NS3, NS4a, NS4b, NS5)^7^. Non-structural (NS) proteins are responsible for the replication of new viruses in the host cell^8^. NS1 can be a reliable biomarker for the diagnosis of infection at the onset stage because it appears at a certain level from the first day of dengue virus infection and becomes undetectable after a few days in the early stage of infection^9,10^.

Several laboratory methods have been employed to monitor dengue infection in the onset period. These include conventional molecular techniques such as enzyme-linked immune sorbent assay (ELISA), reverse transcription-polymerase chain reaction (RT-PCR), and nucleic acid sequence-based amplification (NASBA)^11^. However, these methods requires highly skilled operators, complex procedure, and fancy equipment^12^. In addition, some commercial detection methods based on colloidal gold-labelled monoclonal antibodies such as IgM, IgG and IgA have been demonstrated to be rapid but less sensitive at the onset of dengue infection because IgM & IgG antibodies prominently develop following the decline of viraemia at 3–5 days and 7–10 days after onset of infection, respectively, during a primary infection^12–14^.

Recently, a large number of studies have used nanomaterials for the development of biosensor to detect infectious diseases because they offer exceptional performance by increasing sensitivities, lowering limit of detection, and allowing detection both in vitro and in vivo^15^. Semiconductor quantum dots (QDs) have been widely used as fluorescent probes in the area of biosensors due to their unique properties including high quantum yield, broad absorption, narrow emission spectra, size-tunable light emission, signal brightness, and resistance to photobleaching^16^. Besides, their high surface area to volume ratio and large biomolecule loading capacity render their use as great potential and powerful tool for biosensor applications^17^. There have been several published works using bioconjugates of QDs applied in the areas of cell labeling, imaging, drug therapy, and biosensing^18^. In biosensing, fluorescent QDs have been successfully integrated into sensing systems to serve as probing or transducing components as single fluorophore or donor–acceptor pair-abased sensing materials^19^. In that approach, a decrease in the fluorescence intensity of QDs which is denoted as fluorescence quenching upon conjugating with biomolecules has been utilized to detect biomolecules because it has been proved to be reliable for the detection of target biomolecules^20^. Most recently, some fluorescence immunosensors based on fluorescence quenching have been applied to detect antigen or proteins at very low concentration with some advantages of good sensitivity, high selectivity, and rapidity^21,22^.

In this study, for the first time to our knowledge, we develop a highly sensitive immunosensor which is able to detect even minimal amount of the NS1 antigen in the early stage of infection. To fabricate the immunosensor, the streptavidin-conjugated quantum dots (QDs/SA) are first conjugated with biotinylated NS1 antibodies (Ab), and the QDs/SA-Ab conjugates are then exposed to the NS1 antigen (Ag) at the concentrations of 1 pM to 120 nM both in phosphate buffer solutions and human plasma serum solutions. Fluorescence quenching results are quantitatively analyzed using the theoretical models such as the Stern–Volmer and the Lehrer equations and discussed in terms of the quenching mechanism.

## Experimental

### Materials

Streptavidin-conjugated quantum dots (QDs/SA) with emission wavelength at 655 nm (Qdot® 655 streptavidin conjugate) were provided by Thermo Scientific. According to the supplier’s information, these QDs/SA have core/shell structure of CdSe/ZnS and the particle size of about 15–20 nm. NS1 glycoprotein antigen (Ag) of recombinant dengue virus was obtained from Abcam in South Korea. The biotinylated polyclonal NS1 antibody (Ab, SAB2702396) of dengue virus, Amicon ultra-0.5 mL centrifugal filter unit (100 kDa), phosphate buffer (pH = 7.4), borate buffer, bovine serum albumin (BSA), Tween 20, and human plasma serum were supplied by Sigma-Aldrich. Deionized (DI) water (18.2 MΩ cm) was used in all the experiments.

### Characterizations

In order to measure the first excitation wavelength and absorption intensities of the QDs after each step of the experiment, a UV–Vis spectroscopy (Lamda 35, Perkin Elmer in the range from 200 to 800 nm, slit 2 nm) was used. The fluorescence spectroscopy (Cary Eclipse G9800A) was employed to measure photoluminescence (PL) intensities of antibody-conjugated (QDs/SA-Ab) and antigen-conjugated QDs/SA-Ab (QDs/SA-Ab-Ag). A field emission scanning electron microscope (FE-SEM, JSM-7610F-PLUS, JEOL) and a field emission transmission electron microscope (FE-TEM, JEM-F200, Multipurpose Electron) were used to observe the structures and morphologies of QDs/SA, QDs/SA-Ab, and QDs/SA-Ab-Ag. The FE-SEM samples were carefully prepared on the surface of a piece of silicon wafer, which was coated with gold and covered with copper place using conductive resin tape. The FE-TEM samples were prepared by suspending the conjugates in phosphate buffer solution at low concentration. A tiny drop of the conjugate suspension was place on a carbon-coated copper grid and dried under vacuum. Zeta-potential (ζ) and hydrodynamic particle size of the QDs/SA, QDs/SA-Ab, and QDs/SA-Ab-Ag were measured using a Zetasizer Nano Series (Malvern Inst. Ltd.,). A ζ-potential cell was field with the conjugate suspension and 3 rounds of 15 runs were performed for a sample. For particle size measurements, the samples were prepared by diluting the conjugate suspension with a factor of 10 in DI water and the samples were sonicated for 30 min to break temporary aggregation before the particle size measurements.

### Formation of QDs/SA-Ab conjugates

The biotinylated polyclonal NS1 antibody (Ab) of dengue was first conjugated with the QDs/SA due to the affinity between streptavidin and biotin molecules. Firstly, a diluted suspension of the QDs/SA was prepared at 30 nM by using 100 µL of phosphate buffer (0.1 M, pH = 7.5). According to the supplier’s information, the QDs/SA had the molecular weight of 1.5–2.0 MDa, and the molar concentration of the supplied QDs/SA suspension was 50 mM. This information was used for the concentration calculation of dilution procedure. At the same time, three Ab solutions with different concentrations of 30, 90 and 150 nM were prepared by using 100 µL phosphate buffer containing 0.1% BSA. Each Ab solution was gently mixed with the QDs/SA suspension and then incubated for 1 h in the dark at room temperature (23 ± 2 °C) in a shaker. After the incubation, the mixture was filtrated with the Amicon ultra-centrifugal filter (100 kDa, Sigma-Aldrich) to remove the unbound Ab. The filtrate was washed three times with phosphate buffer containing 0.01% Tween 20 using the 100 kDa cutoff tubes. The QDs/SA-Ab conjugates were then finally suspended in 100 µL phosphate buffer and stored in the dark at 4 °C before further use.

### Quantitative analysis of NS1 antigen in phosphate buffer

**Scheme 1:**
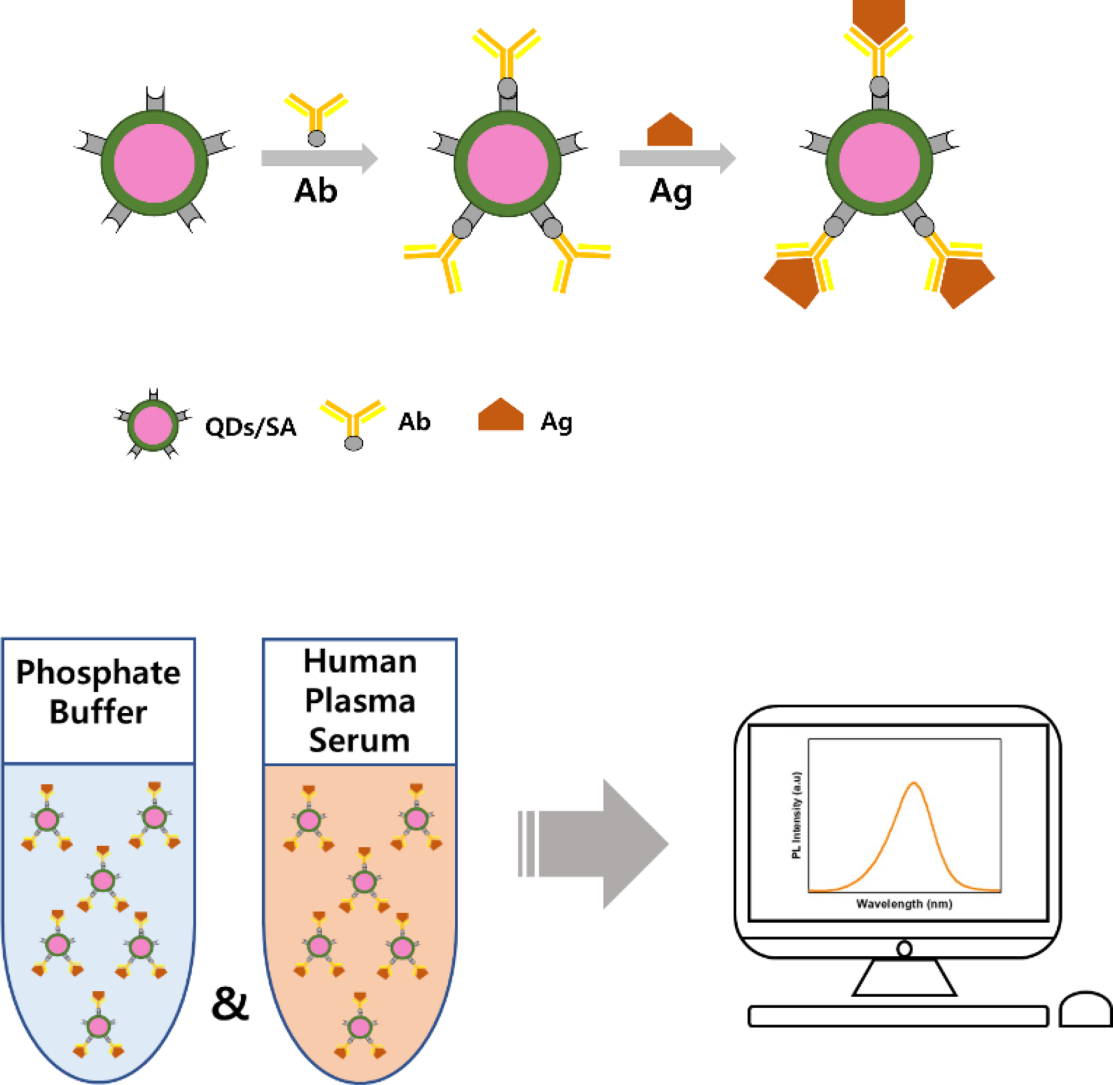
Overall procedure of quantitative analysis of dengue NS1 Ag.

Overall procedure to analyze the NS1 antigen (Ag) quantitatively is illustrated in Scheme 1. Twelve NS1 Ag solutions with different concentrations from 0.1 pM to 120 nM were prepared by diluting in phosphate buffer of 100 µL and they were mixed with the same volume of the QDs/SA-Ag conjugate suspensions. The mixtures were then incubated for 30 min at room temperature (23 ± 2 °C). The photoluminescence (PL) emission spectra of the sample suspensions were measured in the range of 400–750 nm, slit 5 nm, with excitation wavelength at 400 nm. The limit of detection (LOD) was calculated by multiplying the standard deviations of blank measurements by three and dividing by the slope of the linear calibration curve. Each experiment for a specific concentration was repeated six times and the best three results were chosen to obtain the calibration curve. The data selection was based on the consistency of results, and three data closest to the average value were selected to give a minimum standard error. In addition, the fluorescence quenching behavior of QDs/SA-Ab and BSA mixture suspensions was examined to confirm the selective binding of QDs/SA-Ab with the Ag. The BSA buffer solutions were prepared at six different concentrations in the range from 0 to 80 nM and they were then incubated with QDs/SA-Ab. Their PL emission spectra were analyzed to evaluate whether they have any significant fluorescence quenching.

### Quantitative analysis of NS1 antigen in human plasma serum

Similarly, quantitative analysis of the NS1 Ag was conducted in human plasma serum solutions. Firstly, twelve test solutions of 100 µL NS1 Ag were prepared by spiking human plasma serum with the NS1 Ag at different concentrations. The serum was diluted 10 times in a phosphate buffer to obtain a solution at a specific working concentration, and the NS1 Ag solution was injected into the diluted serum solution at different concentrations from 1 pM to 80 nM (100 µL for each concentration). Secondly, the mixed serum suspension was mixed with QDs/SA-Ab suspension of 100 µL and the mixture was incubated for 30 min at room temperature in the dark, and their PL emission spectra were analyzed to evaluate their fluorescence quenching behavior.

## Results and discussion

### Formation of QDs/SA-Ab and QDs/SA-Ab-Ag conjugates

**Figure 1:**
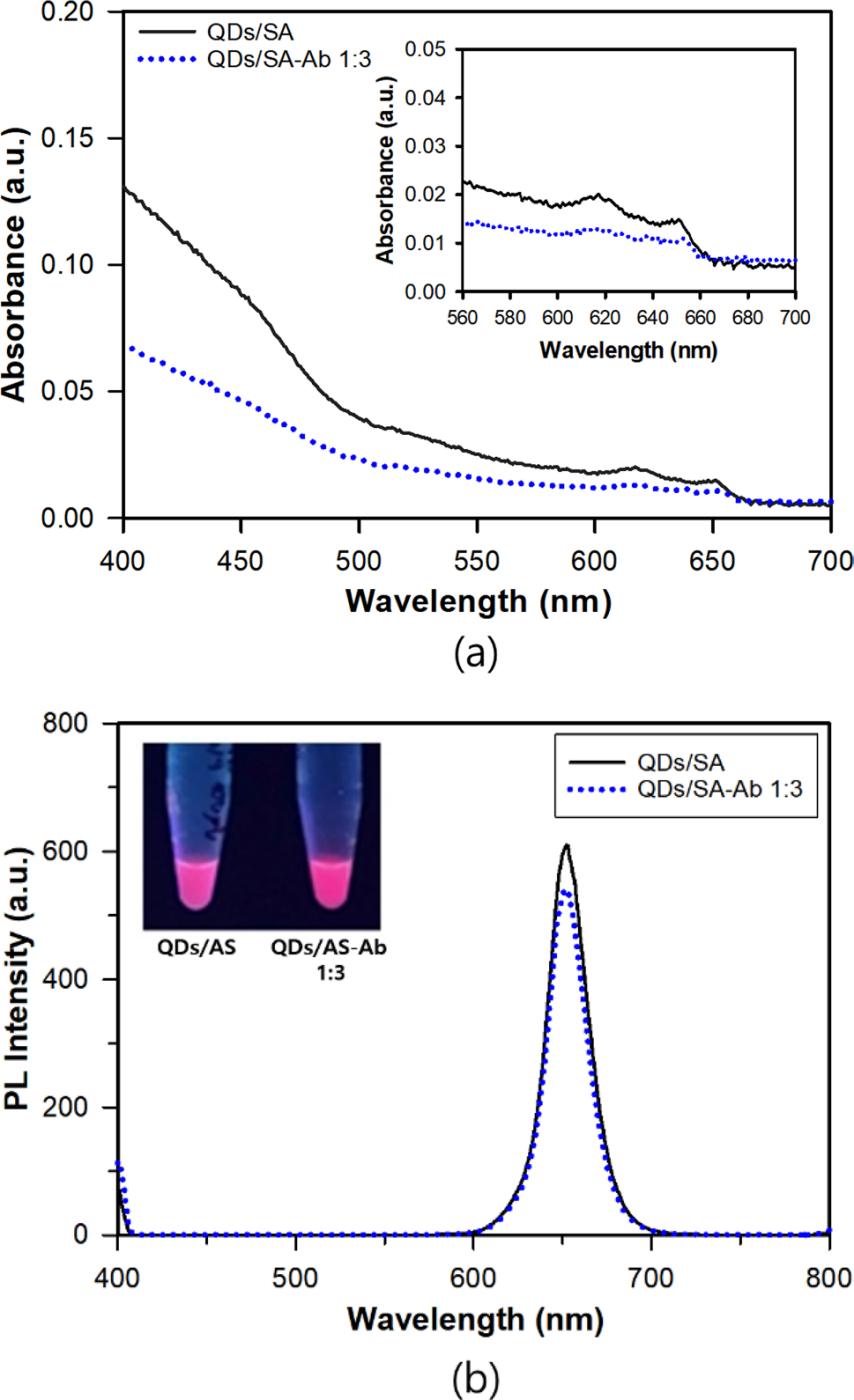
(**a**) UV absorbance and (**b**) PL emission spectra of QDs/SA and QDs/SA-Ab at the ratio of QDs/SA:Ab, 1:3.

First, the QDs/SA was conjugated with the Ab at three different QDs/SA:Ab molar ratios of 1:1, 1:3 and 1:5 in the phosphate buffer solution to check the most appropriate molar ratio. Figure 1a showed the absorption spectra before and after the conjugation, in which two red shifts were observed: one from about 617 to 618 nm for the first excitation and the other from about 651 to 653 nm for the emission. The slight change in absorption spectra may be associated with the change in turbidity in the sample. The absorption spectra were similar shapes before and after the conjugation, which implied that the surface conjugation did not affect the functionality of the QDs/SA nanocrystals^23^. The conjugation of QDs/SA with Ab is known to be high affinity bindings between streptavidin molecules on QDs and biotin molecules on the antibody due to hydrogen bonding and van der Waals interactions^24,25^. As shown in Fig. 1b, the conjugation caused fluorescence quenching, i.e., a decrease in the fluorescence intensity and the magnitudes of quenching were about 2.3%, 11.1% and 54.0% for the molar ratios of 1:1, 1:3 and 1:5, respectively. Because the molar ratio of 1:1 gave too small amount of quenching whereas that of 1:5 did too large amount of quenching, the molar ratio of 1:3 was selectively used. In general, the fluorescence quenching of QDs is known to happen when electron–hole pair recombination is blocked due to the interaction between surface atoms of QDs and molecules or atoms in the medium. The variation of surface or environment of QDs can hamper the electron–hole recombination and decrease fluorescence intensity^26^. The fluorescence quenching of QD is known to be result of photoinduced electron transfer from an exited QD to a proximal molecule with an intermediate in energy to the valence and conduction band edge states^27^. The fluorescence quenching upon the conjugation of QDs with other biomolecules such as antibody and antigen has been reported in the literature^28,29^.

**Figure 2:**
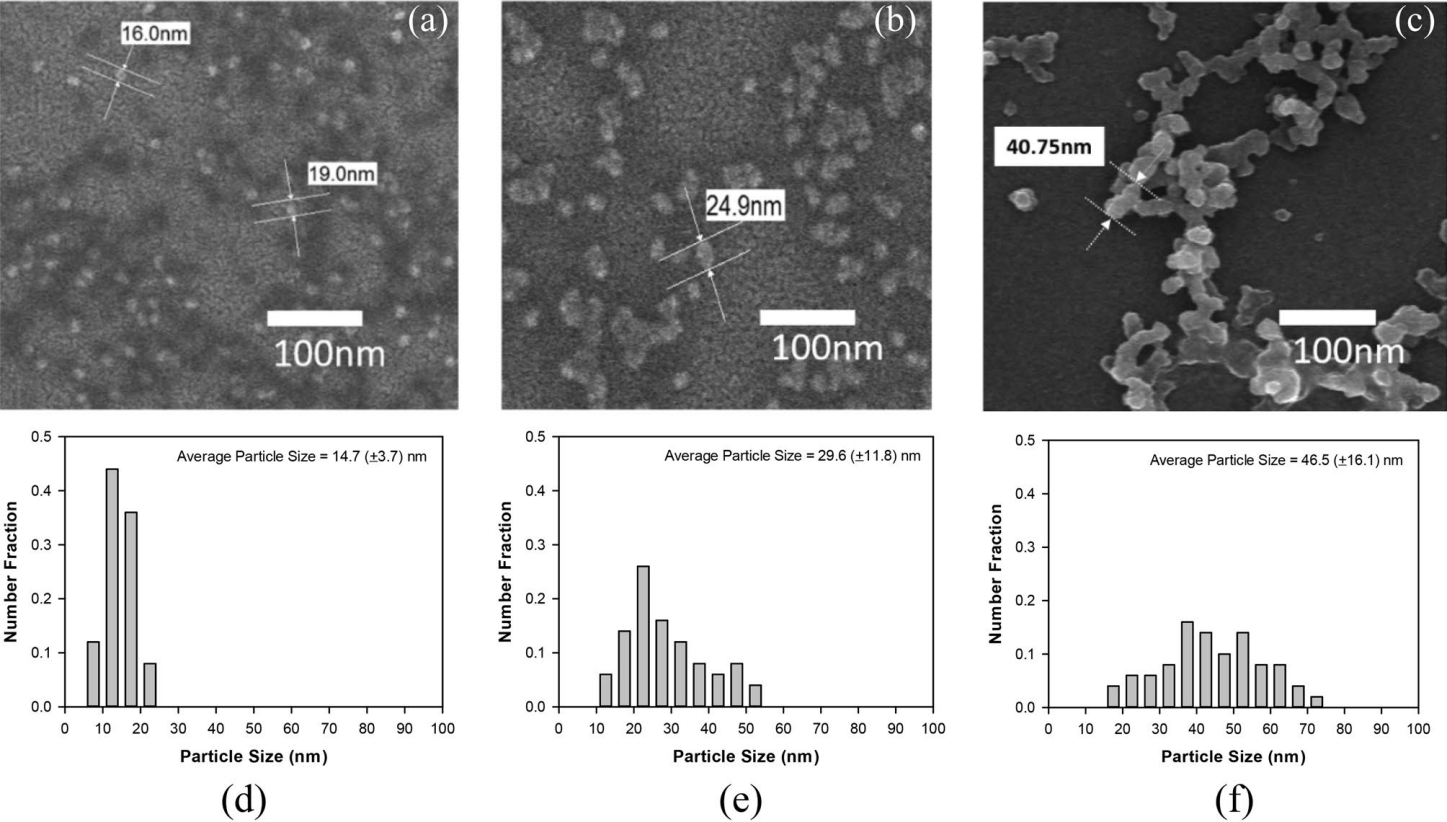
FE-SEM images of (**a**) QDs/SA, (**b**) QDs/SA-Ab, (**c**) QDs/SA-Ab-Ag, and particle size distributions of (**d**) QDs/SA, (**e**) QDs/SA-Ab, and (**f**) QDs/SA-Ab-Ag.

**Figure 3:**
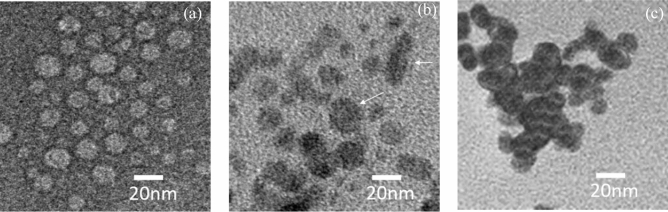
FE-TEM images of (**a**) QDs/SA, (**b**) QDs/SA-Ab, (**c**) QDs/SA-Ab-Ag.

**Figure 4:**
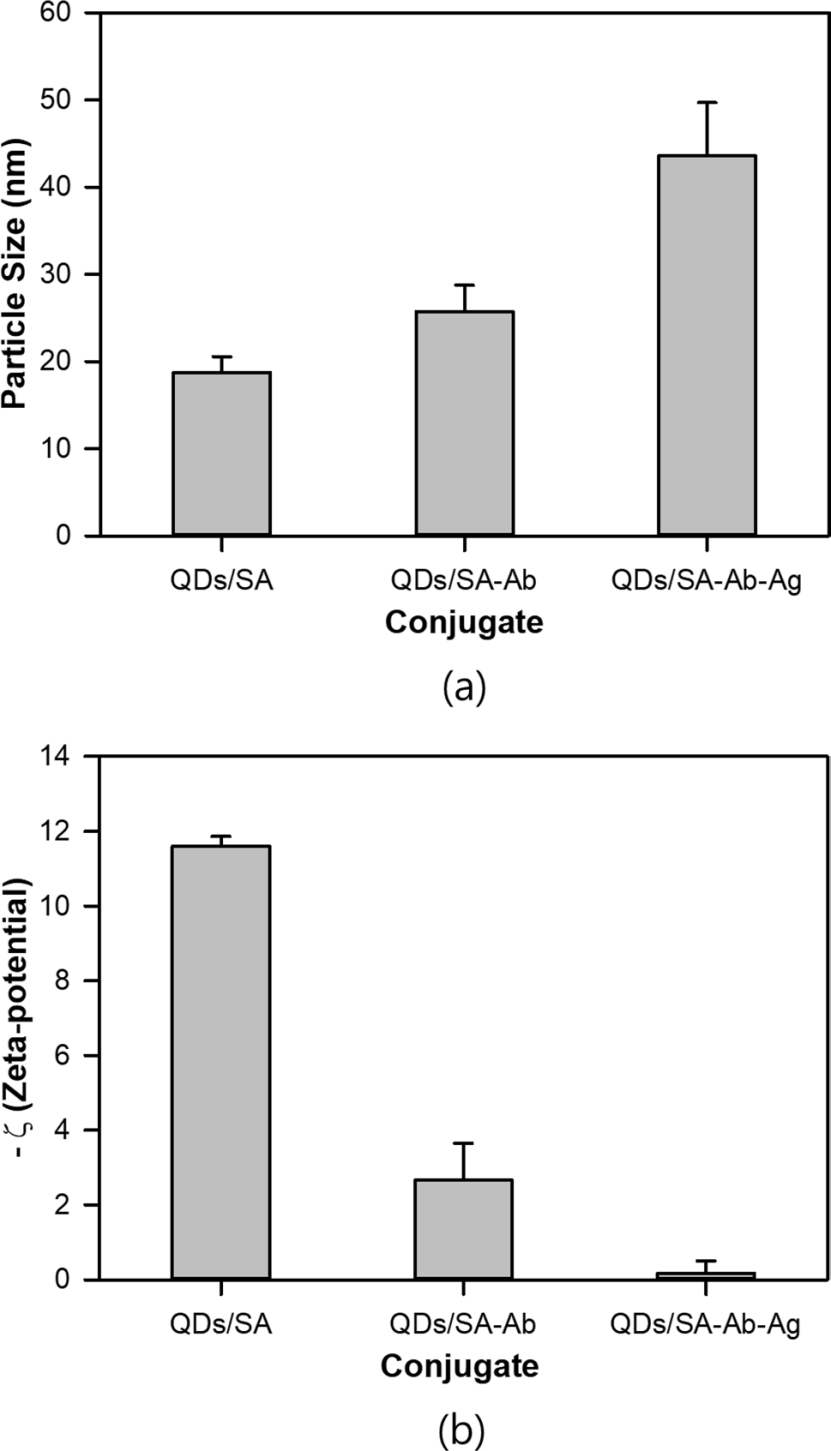
(**a**) Hydrodynamic particle size of QDs/SA, QDs/SA-Ab, QDs/SA-Ab-Ag, (**b**) zeta potential of QDs/SA, QDs/SA-Ab, QDs/SA-Ab-Ag.

In order to confirm the conjugations of QDs/SA with Ab and QDs/SA-Ab with Ag, the structures and morphologies of the QDs/SA, QDs/SA-Ab, QDs/SA-Ab-Ag were observed with FE-SEM and FE-TEM. Figures 2 and 3 show the SEM micrographs and its particle size distribution which was measured for 100 particles arbitrarily selected in two micrographs, and TEM micrographs in which the particle size distribution was not evaluated because the resolution was not enough to evaluate particle size accurately. As shown in Fig. 2, the average particle size of QDs/SA was 14.7 (± 3.7) nm and it increased to 29.6 (± 11.8) nm upon the conjugation with Ab and to 46.5 (± 16.1) nm upon the conjugation with Ag. This increase in particle size was also confirmed by the FE-TEM micrographs in Fig. 3 and by the hydrodynamic particle size measurement results in Fig. 4a. The significant size increase upon the conjugation of QDs/SA-Ab with Ag can be attributed to the particle agglomeration often caused by the bindings between Ab and Ag molecules^30^. For the morphologies, the QDs/SA nanoparticles were apparently spherical in shape and they seemed to become oval shape after the conjugation with Ab. As shown in Fig. 4b, the zeta-potential values of QDs/SA, QDs/SA-Ab and QDs/SA-Ab-Ag were measured to be − 11.6 mV, − 2.6 mV and − 0.17 mV, respectively. For the zeta potential analysis, the pHs of the samples were about 7. The measured zeta potential value of QDs/SA in the similar condition to the current study was consistent with that reported in the literature work^31^. It is presumed that the magnitude of zeta potential decreased upon the conjugation with Ab and Ag because the Ab and Ag molecules cover the charged surface of the QDs/SA particles to form larger aggregates, which intervene the electrical double layer. Although the QDs/SA-Ab conjugate was not expected to have a long-term stability because of its zeta potential close to zero, its suspension was apparently stable after the storage for 4 weeks in the dark by showing no significant aggregation and change in the UV and the fluorescence spectrums. Similarly, the QDs/SA conjugates with biomolecules have been reported to be stable even after 3 months in the literature^32^. Nevertheless, the detection experiments in the current study were conducted within a couple of days after its preparation to minimize stability problem. For further development like a commercialization of the current sensor, the shelf-life of the QDs/SA-Ab conjugate needs to be determined and extended by a proper method such as a surfactant system.

### Quantitative analysis of NS1 antigen in phosphate buffer

First, the detectability of fluorescence quenching was investigated at different concentrations of the QDs/SA-Ab and the Ag solutions to decide appropriate the QDs/SA-Ab concentration for quantitative analysis of Ag. When the magnitude of fluorescence quenching was larger the standard error of control sample, it was considered as detectable, whereas it was not detectable when it was smaller than the standard error. As summarized in Table 1, at the Ag concentration of 0.1 pM, no significant fluorescence change was observed in the PL emission spectra even at the QDs/SA-Ab concentration of 7 nM. However, at the Ag concentration of 1 pM, the fluorescence quenching was detectable at the QDs/SA-Ab concentrations larger than 5 nM. At the Ag concentration of 10 pM, the fluorescence quenching was detectable at all the QDs/SA-Ab concentrations larger than 1 nM. Because this study targeted at the lower detection limit of 1 pM Ag, we decided to use the QDs/SA-Ab concentration of 5 nM, which can detect all the Ag solutions at the concentrations above 1 pM.

**Table 1:** Comparison of fluorescence intensity of QDs/SA-Ab-Ag with that of control sample (no Ag) and detectability of fluorescence quenching at several different concentrations of QDs/SA-Ab and Ag.

[Ag]	[QDs/SA-Ab]			
	1 nM	3 nM	5 nM	7 nM
Control (no Ag) (standard error)	45.3 (1.1)	127.5 (2.6)	163.3 (2.9)	240.8 (5.7)
0.1 pM	45.2 No	126.1 No	162.5 No	236.8 No
1 pM	44.4 No	126.0 No	160.1 Yes	232.3 Yes
10 pM	42.9 Yes	123.8 Yes	157.9 Yes	226.7 Yes

Yes: fluorescence quenching was detectable.

No: fluorescence quenching was not detectable.

The PL emission data of all the samples were measured at 653 nm.

**Figure 5:**
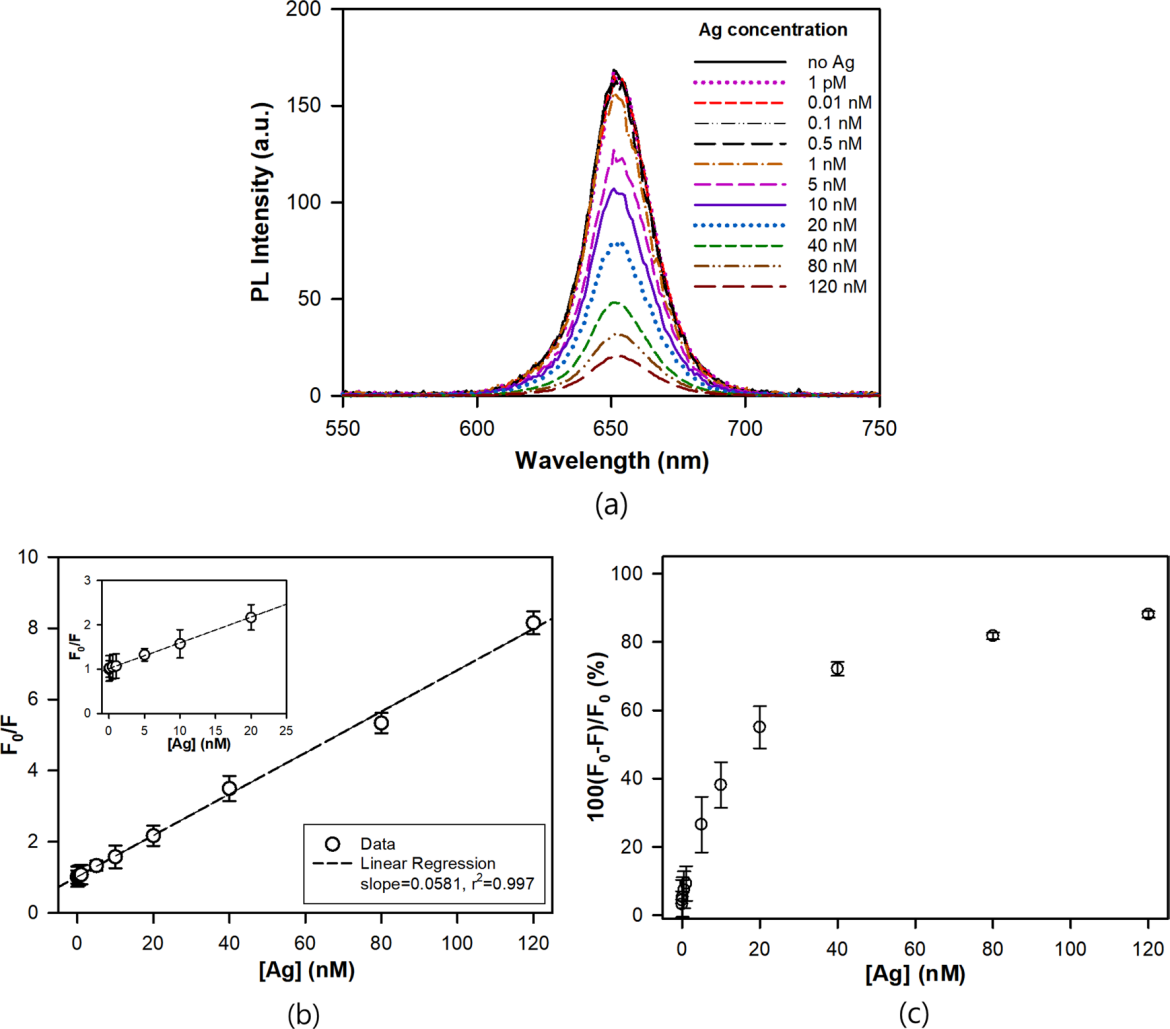
(**a**) PL emission spectra, (**b**) Stern–Volmer plot, and (**c**) quenching efficiency upon conjugation of QDs/SA-Ab with Ag at different Ag concentrations from 0.001 to 120 nM.

As previously stated, twelve Ag solutions in phosphate buffer with different concentrations from 0.1 pM to 120 nM were incubated with the QDs/SA-Ab at the concentration of 5 nM, and their PL spectra were measured. Figure 5 shows the PL spectra for the phosphate buffer solutions of QDs/SA-Ab and QDs/SA-Ab-Ag at different Ag concentrations, and the plot of F_0_/F versus Ag concentration, where F_0_ and F are the fluorescence intensities of QD/SA-Ab before and after binding with the Ag, respectively. As shown in Fig. 5a,b, the magnitude of fluorescence quenching (F_0_/F) significantly increased with the increasing Ag concentration, and it was linearly proportional to the Ag concentration. This relationship can be analyzed by the following Stern–Volmer (SV) equation^33^:

1 $$ {\text{F}}_{0} /{\text{F }} = { 1 } + {\text{ K}}_{{{\text{SV}}}} \left[ {{\text{Ag}}} \right] $$

where K_SV_ and [Ag] are the Stern–Volmer quenching constant and the Ag concentration, respectively. The K_SV_ value for the QD/SA-Ab-Ag complex, which can be defined as sensitivity, was evaluated to be 0.0581 (± 0.001) nM^-1^ (0.359 mL/ng) from the linear data regression (r^2^ = 0.997). There are three mechanisms of fluorescence quenching; static quenching, dynamic quenching and Föster resonance energy transfer (FRET). In the static quenching, the fluorescence intensity is reduced by a formation of nonfluorescent ground state complex, whereas it occurs when the quencher diffuses to the fluorophore making contact in the dynamic quenching. In the FRET, a donor molecule in its electronically excited state transfers its energy to acceptor molecule through non-radiative dipole–dipole coupling^33,34^. The fluorescence quenching mechanism can be deduced from the shape of the SV plot; a good linearity indicates predominant dynamic quenching, an upward curvature (positive deviation from SV plot) means an additional static component, and a downward curvature (negative deviation from SV plot) appears when a fluorophore fraction is not accessible to the quencher^33,34^. Therefore, the quenching mechanism in the phosphate buffer solution was presumably judged to be dynamic quenching from the good linearity of the SV plot^34,35^.

The limit of detection (LOD) and limit of quantitation (LOQ) for the Ag were estimated to be 0.85 (± 0.015) pM (0.041 ng/mL) and 2.83 (± 0.05) pM (0.136 ng/mL), respectively, by using the equations, LOD = 3σ/K and LOQ = 10σ/K, where σ and K are the standard deviation (n = 10) of the blank solution and the slope of line, respectively^20,36^. It is noted that a large error in the slope of line (K) can cause a large error in the LOD and the LOQ. For example, the standard error of 20% in the K value would cause a similar amount of variations in the LOD and the LOQ. In the current work, the small standard error of K value of 1.7% only resulted in small variations of ± 0.015 pM and ± 0.05 pM in the LOD and the LOQ, respectively.

According to theoretical analysis, there are two types of fluorescence quenching, i.e., static quenching and dynamic quenching. The static quenching usually happens due to the ground-state complex formation while the dynamic one is caused by collisions of fluorophores and quencher in the excited plate. The magnitude of fluorescence quenching can be also described using percent quenching efficiency which is defined as 100(F_0_ − F)/F_0_. As shown in Fig. 5c, the quenching efficiency decreased and approached to a plateau as the Ag concentration increased. The fluorescence quenching reached more than 50% and about 90% at the Ag concentration of 20 nM and 120 nM, respectively.

**Figure 6:**
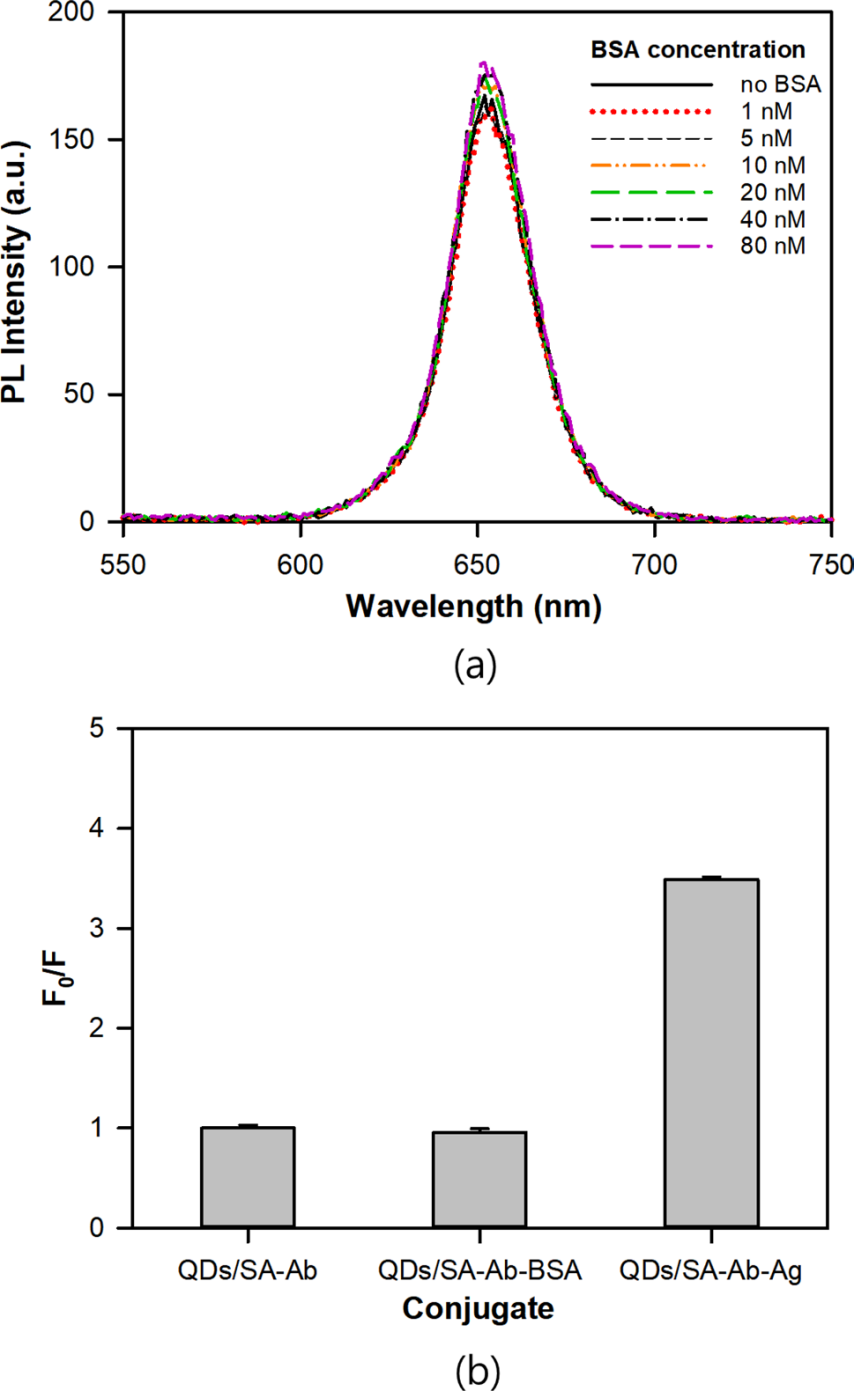
(**a**) PL emission spectra of QDs/SA-Ab upon conjugation with BSA, and (**b**) Comparison of F_0_/F values of QDs/SA-Ab-BSA and QDs/SA-Ab-Ag at concentration of 40 nM BSA or Ag.

Emission spectra of mixture of QDs/SA-Ab and bovine serum albumin were investigated to confirm whether the fluorescence quenching was caused by the selective binding of QDs/SA-Ab to Ag. As shown in Fig. 6a, no significant fluorescence quenching was observed when the QDs/SA-Ab was incubated with BSA at concentrations up to 80 nM in the phosphate buffer solution. As compared in Fig. 6b, the fluorescence quenching of the mixture with BSA was even slightly negative (F_0_/F value less than 1) within error bound whereas that with Ag was more than 70% (F_0_/F value of 3.5). Therefore, the fluorescence quenching apparently resulted from the selective binding of QDs/SA-Ab to Ag.

### Quantitative analysis of NS1 antigen in human plasma serum

**Figure 7:**
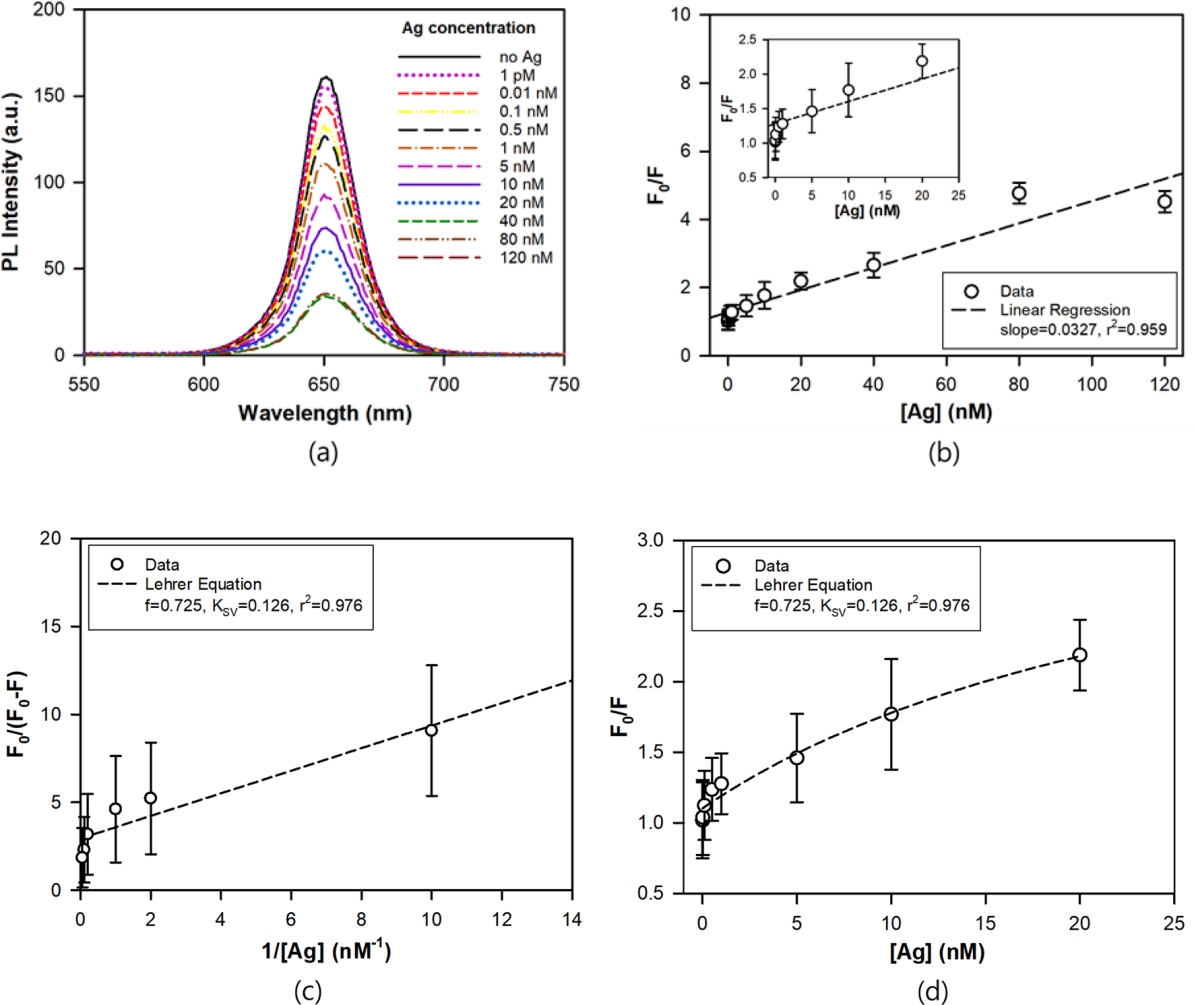
(**a**) PL emission spectra, (**b**) Stern–Volmer plot of QDs/SA-Ab-Ag at different Ag concentrations from 0.001 to 120 nM in the human plasma serum, (**c**) the Lehrer equation plot of F_0_/(F_0_ − F) versus 1/[Ag], (**d**) the Lehrer equation plot of F_0_/F versus [Ag] up to Ag concentration of 20 nM.

The fluorescence quenching of the QDs/SA-Ab-Ag conjugate in human plasma serum solutions was quantitatively analyzed in the Ag concentration range of 1 pM to 120 nM to check an applicability of this approach in practical detection of infection. As shown in Fig. 7, significant fluorescence quenching was observed in the concentration range, and the magnitude of fluorescence quenching (F_0_/F) was approximately proportional to the Ag concentration with a slope (K_SV_) of 0.0327 nM^−1^ (0.202 mL/ng) and a little worse linearity (r^2^ = 0.959) than in phosphate buffer. However, in the lower range of Ag concentration, the relationship between F_0_/F and Ag concentration was quite deviated from the linearity, and it showed a negative deviation from SV plot, which indicated that some parts of fluorophores are not accessible by the quencher. In this case, the data can be analyzed using the following Lehrer equation^34,37^:

2 $$ {\text{F}}_{0} /\left( {{\text{F}}_{0} - {\text{F}}} \right) \, = { 1}/{\text{f }} + { 1}/\left( {{\text{f K}}_{{{\text{SV}}}} \left[ {{\text{Ag}}} \right]} \right) $$

where f is the fraction of accessible fluorophores. As shown in Fig. 7c, the f of 0.725 and the K_SV_ of 0.126 were obtained by the linear curve fitting of the fluorescence quenching data up to 20 nM by the above Eq. (2). In addition, Fig. 7d shows that the Lehrer equation gives a better nonlinear curve fitting of F_0_/F versus [Ag] than the SV plot. The f value means that about 27% of fluorophore was not accessible to the Ag molecules and the K_SV_ value much higher than that by SV plot over the whole concentration range indicated that this approach is more sensitive in lower Ag concentration. This type of negative deviation due to the limited access of quencher to the fluorophore has often been reported with biomolecule fluorophores such as human serum albumin^35^ and fluorescing tryptophan in ovalbumin^38^. These results suggest that in the human plasma serum solution the access of Ag (quencher) to the fluorophores of QDs-SA-Ab is presumably hindered by the component biomolecules (mostly albumins & globulins). However, even with a negative deviation from SV equation, an accurate quantitative analysis was possible using the Lehrer equation in the Ag concentration range of 1 pM to 120 nM, (0.048–5,760 ng/mL). The detectable concentration range in this study is enough to cover the clinical concentration range of NS1 Ag (40–2,000 ng/mL) in the human plasma serum^39^. In the human plasma serum, the LOD and the LOQ were evaluated to be 1 pM (0.048 ng/mL) and 3.33 pM (0.16 ng/mL), respectively. In Table 2, this LOD value was compared with those of similar studies in the literature, and it is much lower than most values reported in the literature.

**Table 2:** Comparison of biosensor platforms and their LOD for dengue virus detection.

Biosensor type	Analyte	Materials	Real sample/spiked sample	Detection range	LOD	Year	Refs.
Immunosensor	NS1	Gold film electrode	Serum	1–100 ng/mL	0.33 ng/mL	2012	^42^
LSPR fiber optic sensor	NS1	Gold Nanoparticles	–	–	1.54 nM	2013	^10^
Immunosensor	NS1	Microspheres	Serum	–	5.2 ng/mL	2013	^43^
Impedimetric biosensor	NS1	Gold electrode	Neat serum	0.01–1.00 μg/mL	30 ng/mL	2015	^44^
SPR biosensors	DENV2 Ag	Gold film	Serum	–	4 × 10^4^ particles/mL	2017	^45^
FITC optical biosensors	NS1	Indium tin oxide	Plasma	15–500 ng/mL	15 ng/mL	2018	^46^
Immunosensor	NS1	Screen-printed carbon electrodes	Spiked serum	1–200 ng/mL	0.3 ng/mL	2018	^47^
LSPR biosensor	NS1	Silver nanoparticles	Spiked blood	0.25–2 μg/mL	0.05 μg/mL	2019	^48^
**Immunosensor**	**NS1**	**Quantum dots**	**Spiked serum**	**0.001–120 nM**	**0.048 ng/mL**	**2021**	**This work**

It has been known that the NS1 protein comprises 352 amino acids residues sharing approximately 70% sequence similarity among four dengue virus serotypes and 40–50% sequence similarity to other flaviviruses such as Zika virus^40^. Therefore, it appears that the detection of NS1 antigen is not associated with a specific dengue subtype and there is a possibility of cross-reactivity of the NS1 antibody to similar biomarkers between flaviviruses such as dengue and Zika. However, a recent study of a retrospective analysis with real time reverse transcription polymerase chain reactions (rRT-PCR) has found that no cross-reactivity of the NS1 antibody to Zika antigen occurs, and thus dengue NS1 antigen assays are entirely proper for dengue surveillance^41^.

In summary, the current results suggest that the immunosensor using QDs/SA may provide a promising tool to detect dengue virus in the early stage because it is highly sensitive, fast, simple, and convenient, and thus it has a potential of application in point-of-care.

## Conclusions

In this work, we fabricated and quantitatively analyzed a novel and highly sensitive immunosensor to detect a typical dengue biomarker, non-structural protein 1 (NS1) antibody (Ag) by employing a fluorescence probe system of streptavidin-conjugated quantum dots (QDs/SA). The conjugation of QDs/SA with biotinylated NS1 antibody (Ab) was successfully completed, which was confirmed by FE-SEM, FE-TEM, DLS and zeta-potential measurements. Quantitative evaluation of the NS1 Ag was successfully conducted in the Ag concentration range of 1 pM to 120 nM both in phosphate buffer and human plasma serum by analyzing fluorescence quenching. The analysis results indicated that in the buffer solution the fluorescence quenching behavior followed the Stern–Volmer (SV) plot which meant the dynamic quenching mechanism, whereas it showed negative deviation from the SV plot which suggested hinderance of fluorophore presumably by biomolecules such as albumins and globulins in the human plasma serum. The limit of detection (LOD) in the human plasma serum was found to be superior by comparing with those reported in the literature. These findings implied that the current approach of using QDs/SA has a great potential of application for point-of-care because of its high sensitivity, simplicity and convenience.
